# Foreign

**Published:** 1842-01-15

**Authors:** 


### FOREIGN.

*Extract from a paper on the treatment of Varicose Veins by the needle and twisted suture.* By T. B. Peacock, Esq., late House Surgeon to the Chester Infirmary, &c.

I was first led to make trial of this plan fiom reading the report of a case by Mr. Melvin, in the number of the London Medical Gazette for July 7th, 1838, and I have since applied it myself, or seen it made use of by the surgeons to the Chester Infirmary, in at least thirty cases, of several of the most important of which I have retained notes. The plan adopted has been that recommended in the paper referred to, of passing a common curved suture needle under the vein, constricting it with a thread in the figure-of-8 form, and having turned the needle on its side, retaining it there by straps of adhesive plaster: at the end of two or three days, the ligature, if only moderately tightened at first, will require to have a fresh one passed over it; and in two or three more the needle may be removed. Several different methods have been proposed for effecting the obliteration of the vein by the needle; but this, which was originally introduced by Velpeau, as being the most simple, is that which I have always adopted. The length of time which it will be necessary for the needle to remain will depend on whether it is intended simply to excite suppuration, or to ulcerate out; the last being the course which I have usually followed, as in one or two instances, in which the needle was withdrawn after exciting suppuration, the obliteration of the vein was found not to have been effected. This plan has, however, been objected to as leaving a sore difficult to heal afterwards; but in only one instance have I seen it attended by any such result. For the needle to ulcerate its way out, the time usually required will be from a week to ten days; but it will vary greatly according to the state of the part in which it is applied: in the immediate neighbourhood of an ulcer, where the skin is thin and inflamed, a day or two will often suffice to commence the ulcerative action, and three or four for the needle to escape; while, when inserted some distance from the seat of disease, and beneath sound integument, the process will require ten days, a fortnight, or even longer. Thus, in a case lately under my charge, where the needle was inserted beneath a tender sinus on the instep, leading to a small ulcer about an inch above, it ulcerated out in three days; while at the same time, in another case, a needle was placed under each saphæna, and one beneath the common vein, at their point of union: the needle on the anterior branch was not removed till the twelfth day, and the other two not till the nineteenth. I have since seen two instances in which the needles were retained till the end of the third week. Generally speaking, when inserted over a bone, they excite ulceration more rapidly than when upon soft parts; and I am inclined to think that, in the last situation, they are more apt to give rise to an undue degree of inflammation: at least, in the only two cases in which their application was followed by troublesome abscesses, they had been inserted beneath sinuses in the calf of the leg. Considerable pain is sometimes excited by the operation, but it usually soon subsides; and I have not, in any instance, known tenderness to extend in the course of the vein above two or three inches from the point of constriction; and in none has it resisted ordinary treatment: indeed, in no instance which I have seen have any serious symptoms resulted from the operation.

The cases in which I have found this treatment applied have been in small irritable sores remaining after the bursting of large varicose sinuses, inveterate ulcers connected with a generally enlarged condition of the veins of the limb, and œdema of the leg and ankle, either simple or attended with a serous discharge from the skin; and in all of the cases but two in which I have seen it had recourse to, the results have been most satisfactory; and in these, as only one needle was inserted, and other sinuses were left unobliterated, success was hardly to be expected. The number of needles which I have generally seen inserted has been three or four in each limb, but, in some instances, five or six have been applied; the rule adopted having been generally to insert in a case of varicose ulcer one under each enlarged vein an inch or so below the ulcer, and again on each trunk a few inches above it, selecting for the points of their insertion the largest sinuses. Sometimes I have adopted the plan mentioned by Mr. Dodd, of placing on each vein two needles an inch or an inch and a half apart, so as to effect adhesion of the sides of the intervening tract; and in these cases the main trunk will, after the cure is effected, be often found contracted to a firm cord up to the point at which the next large vein communicates with it; while, where a single needle only is inserted, the portion of the sinuses around is often not affected by the operation.

The effect produced on the sore by the obstruction to the course of the large veins in connection with it, is often most rapid; the inflamed margin gradually subsides, the edges become depressed, granulations spring up, and cicatrization quickly proceeds; and sores which have been liable to bleed entirely lose that tendency, the granulations becoming firm. I have, however, observed what has been noticed before by Mr. Dodd, that the healing process was not equally rapid throughout, the good effect produced by the needles sometimes gradually subsiding, and considerable difficulty being experienced in obtaining the entire healing of the sore.

In this way ulcers which had long been under treatment, without deriving any advantage, have, in several instances, been cured, and others which were found to return as soon as the patient resumed his work, have, by the aid of a laced stocking, been kept healed; indeed, not only does it appear to be a rapid method of effecting the cure of these cases, but I am inclined to regard it as also a more permanent one. The first case in which I made trial of the practice was one of œdema of both legs, attended with excoriation of the skin, and a fœtid discharge, connected with a very varicose state of the large veins. The man, by trade a rope-maker, had been repeatedly under treatment before with very partial benefit; and no sooner did he resume work than the disease returned. On this occasion he had been subjected to the ordinary treatment during a month that he had resided in the infirmary, but with little or no advantage. Under these circumstances, as the case seemed to offer a fair opportunity for treatment with the needles, three were inserted beneath large sinuses in one leg, which was nearly well before the same plan was adopted in the other. He was discharged, entirely cured, on needles being introduced in the other limb, in six weeks from the commencement of the treatment. Two years have now elapsed, and he continues perfectly free from any return of his complaint.—Of two men one had suffered from varicose ulcers on both legs for nine years, the other for five; and both had been several times under treatment in neighbouring infirmaries, but no sooner did they return to their work, that of cotton-spinning, than the ulcers again broke out. Seven needles were inserted in the legs of one, and three in the other; and both were cured, one in seven, the other in three weeks, and continued so for at least four months, during which I had an opportunity of noticing them. Indeed the absence of any pain, swelling, or weakness in the limbs, which they said, as healed before, they had always found to continue, and the sound appearance of the cicatrices, afforded a fair prospect of permanent cures having been effected. The state of the limb afterwards, and the pale, healthy-looking cicatrices, form a great contrast between cases treated by this and by the ordinary methods. I had a case recently under my charge, in which an ulcer, fully the size of the palm of the hand, was entirely cured in little more than a month, and this notwithstanding that copious suppuration was excited by the needles in the cellular membrane of the calf of the leg. This patient had previously been subjected to treatment for four months, with every advantage of circumstances for the cure of a sore in the same situation; and the case was further interesting as being attended by severe pain in the sole of the foot—an occurrence which was met with in one of Mr. Dodd's patients—and having been an old man of 70; while Bonnet, in an essay on this subject, published in Paris, has stated that the operation will not be successful after the age of 60, in consequence of the indisposition of the blood to coagulate, and that it should not be attempted. I heard of the man several months after his discharge; he was following his work and his limb continued sound. I regret that, in consequence of most of the patients on whom the plan was tried in the infirmary residing at a distance, I am not able to speak of them after they left the institution.

The above remarks were written more than twelve months ago. I

have now nothing further to add than that additional experience fully confirms the opinion expressed of the safety and rapidity of the cure of disease dependent on varicose veins, by the plan referred to, and I have reason to regard it as also a permanent one, care being of cours- taken to support the limb by a laced stocking or bandage, as other, wise the same cause which gave rise to the varicose condition of the veins will lead to the dilatation of fresh ones.—*London Med. Gaz. Nov.* 5, 1841.

---

*Hyper-operating*.—The Lancet again brings before us an extract from the paper of Mr. J. Braid, published in the Edinburgh Medical and Surgical Journal. It is a favour to the reviewer when the original writer spares him all necessity for seversty in criticism, by *a clearness of style and a decision both of purpose and expression* which render comment supererogatory. We give the reader two short extracts from the paper in question; simply italicising a few words, and adding two notes of *admiration*.

"Different instructions as to what tendons should be divided in each variety of varus are given by surgeons, and I find an American editor, Dr. Chase, is advocating mechanical extension alone. His cases comprise no difficulties, and would have been cured in less time, and with less pain, by the cutting treatment.

Having operated upon two hundred and forty-six club-feet, embracing every variety and every age, from fifty-three years to two days old; and having, in the course of this practice, tried many modes of operation, the results of which I have watched with strict attention, I trust I may not be deemed presumptuous in offering opinions different from others who have written upon the subject, *but whose opportunities of observation may not have been so ample as my own*, however competent they might have been in other respects.

It appears to me, that it is not enough to say that any given method should be adopted, because cases have been cured by that plan. The plans must vary according to the cases.

In a case of pure talipes equinus with rigid contraction, it will generally be found that not only is the tendo Achillis and plantaris implicated, but also the flexor longus pollicis pedis and flexor longus digitorum, tibialis posticus and peronæus longus and brevis. I am aware that after dividing the tendo Achillis alone, forcible extension will be adequate to bring the foot into its natural position; but I am certain that it will be after much longer time has been expended, than if all the rigid muscles had been divided at once. Moreover, I confidently affirm that if all are divided, and the foot brought to its natural position *within four or five days*, sufficiently firm reunion will have taken place to enable the patient to extend the foot. If any of these muscles are relaxed, it would be superfluous to divide them; but as they are generally proportionally contracted, by dividing them, the patient is saved much suffering during the extension, and the danger of relapse is lessened.

The wounds being closed with adhesive plaster, the foot and leg should be bandaged as high as the knee, to retain the foot in its original malposition for a few days, and absolute quiet and rest should be enjoined. Extension should begin in two days, and in three or four the foot may be brought to a right angle with the leg.

For the same reason in varus, I divide at once every tendon which can possibly retain the foot in its malposition; viz., Achillis, tibialis anticus, tibialis posticus, flexor longus pollicis pedis, and flexor longus digitorum, and abductor pollicis, if required; and should there be much contraction of the sole of the foot, the plantar fascia as well as the short flexors! The wounds being closed, and the foot maintained in its original position, in two days extension may be commenced, so as to bring down the heel, and evert and flatten the foot at the same time. I consider the treatment by *successive* divisions of the tendons as objectionable. 1st, it retards the cure; 2d, it causes too much new tendon to be inserted between the divided ends of the tibialis anticus, and thus weakens the power of bending the foot; and, 3d, it renders necessary a second division of the tibialis posticus, flexor longus pollicis pedis, and flexor longus digitorum.

In valgus the peronæus longus and brevis generally require division, and sometimes also the tendo Achillis, peronæus tertius, and extensor longus digitorum, and proprius pollicis pedis. In two days such splints as will maintain necessary extension should be applied.

In calcaneous talipes the tibialis anticus, extensor proprius pollicis pedis, extensor longus digitorum pedis, and peronæus tertius, generally require to be divided. Divide *either all or as many as seem to bear on maintaining the malposition!*

We cannot quit this subject without recommending to the notice of some of our American brethren of the knife, the care with which the muscles so unhesitatingly divided, almost *ad libitum*, are *named* by the author of the paper. Some of our operators have been contented with merely *numbering them*, as thus: *I divided three, five, or six tendons*—as the case may be—*and bronght the heel down in three, four, or five days.* If this method be really preferred, after due consideration, it might be well to publish a stereotype formula with the necessary blanks, as the multitude of operations in this line is increasing to such an extent that it will soon become a distinction in surgery *not to be a distinguished tendon cutter.*

---

*Treatment of Asphyxia from Drowning.*—As most practitioners in the city or country are occasionally called upon to attempt the resuscitation of persons apparently drowned, yet not so frequently as to bear in mind at all times the differences of opinion among the more experienced, as to the artificial respiration, and the time at which it becomes useless, we extract the following remarks from a debate on the subject before the Westminister Medical Society, in October last, as published in the London Lancet for October 30th.

Mr. Forbes Winslow led off this portion of the debate, which was collateral with a discussion on the various forms of asphyxia in new-born infants, eliciting nothing new, as far as we can judge from the report.

"Mr. Winslow was of opinion that inflation by air breathed from the mouth of the practitioner was the best plan of carrying on artificaal respiration. Experiments had shown that the air so used was but little altered in quality, and contained only one-hundredth part less of oxygen than it did previous to its being respired. With respect to the recovery from asphyxia by drowning, Dr. A. T. Thomson had related a case in which a person who had been twenty minutes under water was restored; while other cases were recorded, in which three minutes' immersion was sufficient to entirely destroy life, and frustrate all efforts for its revival. How did we account for this discrepancy ? The most experienced pearl-divers could not remain under water more than five minutes. He (Mr. Winslow) thought that when persons fainted immediately on submersion, vitality was longer retained in consequence of there being less demand upon the system than when struggles were made by the sufferer."

"Dr. Chowne would look with suspicion on any case of recovery from asphyxia by drowning, in which it was stated that the person had been under water for more than six or seven minutes. He believed, indeed, that there was no well-authenticated case of recovery in which the submersion had exceeded six minutes."

"Mr. Wooley thought Mr. Read's instrument would be of great service in asphyxia, whether occurring in adults or infants. He had been in the habit of using the means recommended by the Humane Society to keep up artificial respiration, for some years. The instruments used were the bellows and the trachea-tube. The use of the bellows might be productive of much injury; and it was not always an easy matter, even to those who knew the anatomy of the parts well, to insert the trachea-tube into the proper orifice. Mr. Read's instrument did away with these objections, and he considered it an admirable means of keeping up artificial respiration." "In asphyxia from drowning, however, he had seldom found much benefit from attempts to keep up artificial respiration. When a body was brought to the receiving-house, it was immediately placed in a bath at 100°. Respiration most frequently occurred spontaneously whilst the person was in the bath, and in some cases it took place before the patient had arrived at the station. If, after the body had been placed in the bath, there was no appearance of vitality, he had generally found the employment of artificial respiration of no benefit. Respiration, when first returning, was generally interrupted and spasmodic; it then became regular, and frequently no ill consequences followed. There was, however, in many cases, much mischief supervening in the form of congestion of the brain and thoracic viscera. In some cases of this kind, the convulsions were so great, that the patient was held with difficulty whilst blood was abstracted. In these cases the only instrument he had found of service was the lancet; and it was often necessary to take away a considerable quantity of blood.

Mr. Woolley then read a case illustrating the length of time during which life may be preserved under water. It was first published in the report of the Royal Humane Society in 1840, but as the report is in few hands in America, we extract from it every thing essential.

"At about half-past ten in the morning, a police-constable on duty, and the gate-keeper, saw a man, a little more than half way over the bridge, place his hat upon the pavement, mount the parapet, and leap into the water. The policeman ran to the spot where the occurrence had taken place, and the gate-keeper to his lodge, in which was one of the society's speaking-trumpets, to use it in calling for a boat; but before doing so, he took out his watch, and carefully observed the time. When he saw the boat coming, he ran and joined the policeman and others who were observing the man in the water. From the time of his jumping in he had remained under the surface, supported in a perpendicular position by the flaps of his coat, which lay *upon* the surface, a small portion of the scalp being out of the water. His face was never above the surface, and a great number of small air-bubbles kept coming up from the commencement. He moved very little—at length a large bubble came up, and he immediately began to sink deeper; the bystanders now exclaimed that it was all over with him. At this moment the boat reached the spot, when he had sunk so low that his coat tails were but just within the reach of the arm of the man employed by the institution, who, however, got hold of them, and pulled him into the boat. The gate-keeper then looked at his watch, and found that exactly *five minutes* had elapsed since he first noted the time. The man was apparently dead; but just after he was laid down in the boat a low moan was heard, another midway between the bridge and the receiving-house, and a third when he was on the stretcher being carried into the house. There was, however, no *appearance* of breathing nor of life when he was put into the warm bath, which was ready upon his arrival; but, almost immediately after, convulsive and irregular breathing commenced—he breathed irregularly and moaned occasionally for about fifteen minutes. In five minutes more I saw him; his breathing was then quite natural, his pulse regular and good; he had no pain in the head or chest, nor any where else, and felt pretty well; his face, however was very pallid, and his eyes suffused: he looked like a man who had suffered from habitual intemperance, which I found was the case. I now had him taken from the bath, and put upon the warm bed, apparently free from cerebral or *thoracic* congestion, and only wanting strength to enable him to go home. As the ward was too hot and close, I desired that a little air might be let in, and left the room for a short time. On my return, I found that my order had been misunderstood, and all the windows had been opened. I was absent only a very few minutes in the adjoining room. The effect of this admission of cold air was to produce oppressed breathing and much cough, and to require treatment and watching, which detained me six hours at the receiving-house; they would not take him into St. George's Hospital and, till the expiration of that time, I did not think it safe to send him home. This shows the importance of warm air in resuscitation."

---

The use of iodide of potassium has become so general of late, that any facts relative to its practical operation are interesting. The following conversation on this subject took place at the London University College Medical Society, Oct. 8th, 1841. The questions in reference to this medicine were—first; whether the occasional injurious effects of it are confined to large or small doses? and secondly; what is the peculiar constitutional state in which unpleasant results are most apt to ensue? The remedy is so valuable that we should be very sorry to lose it; and we would be glad if our correspondents would notice as much as possible the favourable or injurious effects of the iodide of potassium, and other preparations of iodine.

*Injurious effects of Iodide of Potassium in small and large doses.*

Mr. Erichsen related a case of chronic rheumatism, in which he had administered five grains of iodide of potassium in two doses, when alarming symptoms came on. There was difficulty of breathing, pain in the chest, excessive discharge from the eyes and nostrils, inflamed conjunctivæ, and most of the symptoms of violent catarrh. The iodide was discontinued, and the patient speedily recovered. He thought the case remarkable on account of the small doses of the iodide that had been given, and the rapidity with which the patient recovered when the medicine was left off.

Mr. Hardwicke had seen even smaller doses than those mentioned by Mr. Erichsen produce the same effects. He thought that these symptoms came on in a peculiar state of the system at the time, and were not the result of a particular idiosyncrasy.

Mr. Bucknill had also seen the effects spoken of from the use of the iodide. It was remarkable that when doses of a scruple or half a drachm were given, the bad effects, when they did take place, were by no means increased in proportion to the dose.

Dr. Lankester had always witnessed the severest effects from large doses. Inflammation of the eyes, erythematous eruptions, and faintings, attended with oppression at the epigastrium, were the most prominent symptoms. These effects yielded quickly to abstinence from the medicine. He did not think large doses increased the absorbent effect of the medicine.

---

*The Medical Examiner* is published every Saturday by *J. C. Turnpenny*, N. E. corner of Spruce and Tenth streets. Terms—*three dollars* a year for one copy, or *five dollars* for two copies sent to the same post office, in all cases in advance. Postage the same as on newspapers. Letters to be addressed,—post-paid,—to the Editors of the Medical Examiner, Philadelphia.
*Reynell Coates, M. D.*, Acting Editor.
*J. B. Biddle, M. D.*, and *W. W. Gerhard, M. D.*, Co-editors.
*J. B. Biddle, M. D.* Proprietor.

